# Zoledronic Acid in a Mouse Model of Human Fibrous Dysplasia: Ineffectiveness on Tissue Pathology, Formation of “Giant Osteoclasts” and Pathogenetic Implications

**DOI:** 10.1007/s00223-020-00752-w

**Published:** 2020-09-01

**Authors:** Alessandro Corsi, Biagio Palmisano, Emanuela Spica, Annamaria Di Filippo, Ilenia Coletta, Michele Dello Spedale Venti, Rossella Labella, Francesca Fabretti, Samantha Donsante, Cristina Remoli, Marta Serafini, Mara Riminucci

**Affiliations:** 1 Department of Molecular Medicine, Sapienza University, Viale Regina 324, 00161 Rome, Italy; 2 Department of Genetics and Development, Columbia University Irving Medical Center, New York, NY USA; 3 Department of Physiology and Cellular Biophysics, Columbia University Irving Medical Center, New York, NY USA; 4 Centro Ricerca M. Tettamanti, Department of Paediatrics, San Gerardo Hospital, University of Milano-Bicocca, Monza, MB Italy

**Keywords:** Fibrous dysplasia, Nitrogen-containing bisphosphonates, Giant osteoclasts, Zoledronic acid, Denosumab

## Abstract

We compared the effects of a nitrogen-containing bisphosphonate (N-BP), zoledronic acid (ZA), and an anti-mouse RANKL antibody (anti-mRANKL Ab) on the bone tissue pathology of a transgenic mouse model of human fibrous dysplasia (FD). For comparison, we also reviewed the histological samples of a child with McCune–Albright syndrome (MAS) treated with Pamidronate for 3 years. EF1α-Gsα^R201C^ mice with FD-like lesions in the tail vertebrae were treated with either 0.2 mg/kg of ZA at day 0, 7, and 14 or with 300 μg/mouse of anti-mRANKL Ab at day 0 and 21. All mice were monitored by Faxitron and histological analysis was performed at day 42. ZA did not affect the progression of the radiographic phenotype in EF1α-Gsα^R201C^ mice. FD-like lesions in the ZA group showed the persistence of osteoclasts, easily detectable osteoclast apoptotic activity and numerous “giant osteoclasts”. In contrast, in the anti-mRANKL Ab-treated mice, osteoclasts were markedly reduced/absent, the radiographic phenotype reverted and the FD-like lesions were extensively replaced by newly formed bone. Numerous “giant osteoclasts” were also detected in the samples of the child with MAS. This study supports the hypothesis that osteoclasts per se, independently of their resorptive activity, are essential for development and expansion of FD lesions.

## Introduction

Nitrogen-containing bisphosphonates (N-BPs) constitute the major class of drugs used to treat various bone diseases including osteoporosis, hypercalcemia of malignancy, Paget bone disease and pediatric disorders with low bone density and increased bone fragility as osteogenesis imperfecta [1, 2]. N-BPs inhibit bone resorption by interfering with osteoclast activity and by promoting osteoclast apoptosis [2]. However, the number of osteoclasts during N-BPs administration may reduce, remain unchanged or even increase [3, 4]. In addition, N-BPs treatment may associate with the appearance of “giant osteoclasts”, large polykaryons with even more than 40 nuclear profiles, compared to 2–8 nuclear profiles found in normal osteoclasts, that are often detached from the bone surface and up to 30% apoptotic [3, 4].

N-BPs are currently used to treat also fibrous dysplasia of bone [Polyostotic FD/McCune–Albright syndrome (MAS); OMIM#174800]. FD is a potentially crippling skeletal disease caused by post-zygotic activating mutations of the alpha subunit of the stimulatory G protein encoded by the GNAS gene (GNAS complex locus; GNAS, OMIM *139320) that can occur either isolated or in association with non-skeletal disorders (mainly hyper-functional endocrinopathies and skin hyperpigmented lesions) in the MAS (for review see [5]). FD lesions are characterized by marrow fibrosis, abnormal bone trabeculae with defective mineralization and increased orthotopic (on bone surfaces) and heterotopic (within the fibrous tissue) osteoclastogenesis [6, 7]. Clinical studies performed with N-BPs in FD patients almost invariably reported a reduction in serum levels of bone turn-over markers, whereas the effects on pain remain controversial [5, 8–15]. In addition, neither a positive effect on the radiographic findings of well-established FD lesions (i.e., refilling/reduction in size, arrest of the expansion) or their tissue pathology, nor significant changes in fracture rate, skeletal disease burden and natural history of FD were definitely established, in particular in pediatric subjects [5, 9, 11].

In contrast, sporadic clinical studies performed with denosumab, a humanized antibody against the central osteoclastogenic factor RANKL, showed that RANKL inhibition may effectively reduce the growth rate of FD lesions, besides reducing pain and bone turn-over markers (for review see [5]). Using the transgenic EF1α- Gsα^R201C^ mouse model of FD [16] and a murine analog of denosumab anti-mouse RANKL antibody (anti-mRANKL Ab), we recently demonstrated that the beneficial effect of RANKL inhibition in FD is underlaid by critical changes in the histopathology of the disease [17]. Indeed, in transgenic FD mice the anti-mRANKL Ab completely suppressed osteoclastogenesis and led to the replacement of the pre-existing FD tissue with a highly mineralized and mechanically sound bone. In addition, it prevented the development of new lesions and the progression of the disease. However, in the same study, we also demonstrated that resumption of osteoclast formation and activity after treatment discontinuation was associated with progressive resorption of the newly formed bone, reappearance of the FD-like tissue and, as in human patients [5], with changes in calcemia, phosphoremia and in the serum level of bone turn-over markers.

As an initial attempt to clarify the reasons for the different effect of N-BPs and anti-mRANKL Ab on the pathology of FD, in this study we treated the EF1α- Gsα^R201C^ transgenic FD mouse model with the N-BP zoledronic acid (ZA). For comparison, we also reviewed the histological samples of a previously published MAS patient treated with an N-BP [18]. We demonstrated that the administration of ZA alone had no significant effect on the tissue changes of FD and was associated with the persistence of intra-lesional osteoclasts and with the appearance of “giant osteoclasts”. We also confirmed that the anti-mRANKL Ab leads to the formation of mineralized bone in the setting of FD lesions [17]. Overall, our results support the hypothesis that osteoclasts per se, independently of their resorptive activity, are essential for development and expansion of FD lesions.

## Materials and Methods

### Mice and Experimental Groups

Generation and characterization of EF1α-Gsα^R201C^ mice was reported previously [16]. The animals were maintained in cabin-type isolators at standard environmental conditions (22 to 25 °C, 40 to 70% humidity) with 12:12 dark/light photoperiod. Food and water were provided ad libitum. All studies were performed in compliance with relevant Italian laws and Institutional guidelines and all procedures were IACUC approved (authorization 209/19-PR from Health Minister, Italy).

Transgenic EF1α-Gsα^R201C^ mice were selected at the age of 3 months based on radiographically detectable FD-like lesions in the tail vertebrae as described previously [17]. Fifteen females were used in this study. Five mice were treated intra-peritoneally with 0.2 mg/kg of ZA [19] at day 0, day 7, and day 14. Of the other ten mice, five were treated with 300 μg/mouse [17] of anti-mRANKL Ab (Clone IK22/5; Bio X Cell, West Lebanon, NH, USA) by intraperitoneal injection at day 0 and day 21 (positive controls) and five were left untreated (negative controls). Mice underwent radiographic analysis at the end of the 3rd week (T21) and immediately after the sacrifice at the end of the 6th week (T42).

### Microradiographic Analysis

Faxitron MX-20 Specimen Radiography System (Faxitron X-ray Corp., Wheeling, IL, USA) set at 24–25 kV for 6–8 s was used for radiographic analysis of the mice. At T21, X-rays were taken under anesthesia with a mixture of Zoletil 20 (Virbac SA, Carros, France) and Rompun (Bayer, Leverkusen, Germany).

### Histology, Tartrate-Resistant-Acid-Phosphatase (TRAP) Histochemistry and Histomorphometry

Mice were euthanized by carbon dioxide inhalation and their tails were fixed with 4% formaldehyde in PBS pH 7.4 for 48 h at 4 °C. The bone samples were decalcified in 10% EDTA-PBS and routinely processed for paraffin embedding. Three-micron-thick sections were stained with hematoxylin and eosin (H&E). TRAP histochemistry was performed using Sigma Aldrich reagents (Sigma Aldrich, Saint Louis, MO, USA) according to the manufacturers’ instructions.

Histomorphometric analysis was performed on at least 15 tail vertebrae per group and five tibiae per group. H&E-stained sections of FD-affected tail vertebrae and unaffected proximal tibiae were used to measure trabecular bone volume over tissue volume [bone volume/tissue volume, BV/TV (%)] and to count the number of osteoclast nuclei (N.nuclei) and the number of apoptotic osteoclasts, recognized based on the presence of chromatin condensation, nuclear fragmentation and peripheral beading and cell shrinkage [3, 4]. TRAP-stained sections were used to assess the number of osteoclasts over tissue area [number.osteoclasts/tissue area, N.Oc/T.Ar (/mm^2^)]. Sections were scanned via Aperio Scan Scope CS (Leica Biosystem Imaging, Nußloch, Baden-Wurttemberg, Germany) and analyzed using the Aperio ImageScope™ program (v12.3.2.8013) according to the guidelines of the American Society of Bone and Mineral Research [20].

### Human FD

For comparison, we reviewed the histological samples of a previously published patient with MAS [18]. Briefly, the patient was treated with an N-BP (Pamidronate, intravenous administration, 1 mg/kg/3-day-cycle every 6 months) starting at the age of four years. The reviewed samples were obtained 3 years later when sub-trochanteric and diaphyseal corrective osteotomies stabilized with a cervico-diaphyseal intra-medullary nail were performed.

### Statistical Analysis

To determine statistical differences among the three experimental groups, one-way ANOVA test with Tukey’s post hoc test for multiple comparisons were used, whereas a t-test was used to compare two groups. Statistical analyses were performed using GraphPad Prism version 8 (GraphPad Software, La Jolla, CA, USA). All tests were two-tailed and a *p*-value less than 0.05 was considered statistically significant. All data were expressed as mean ± SEM.

## Results

### Microradiographic Analysis

**Fig. 1:**
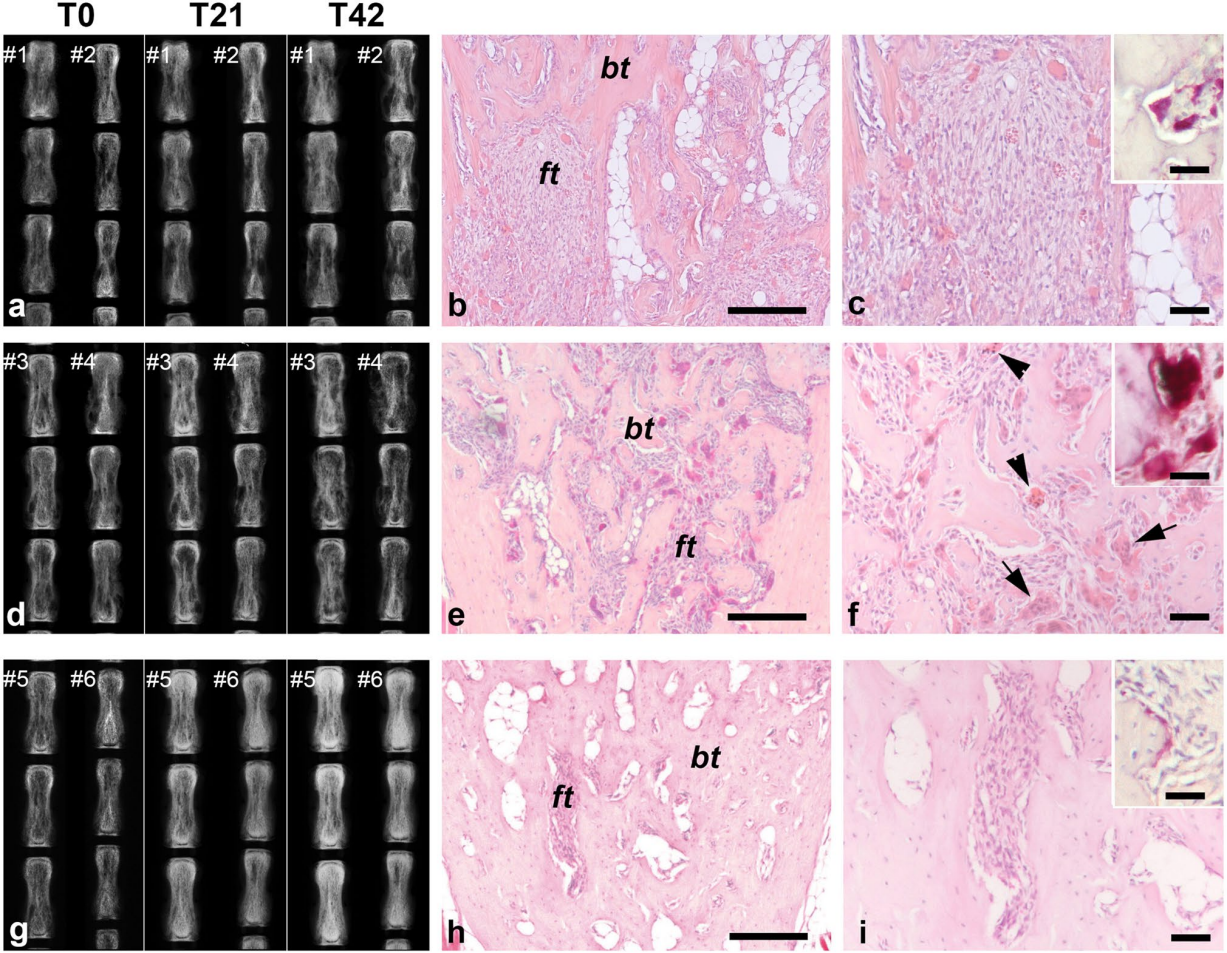
Representative radiographic and histological views of tail vertebrae from untreated mice (**a**–**c**), mice treated with ZA (**d**–**f**) and mice treated with anti-mRANKL Ab (**g**–**i**). Comparative radiographic evaluation of the vertebrae of two representative mice for each group demonstrates the absence of relevant differences between untreated mice and mice that received ZA. However, in some vertebrae of ZA-treated mice (#3), a subtle gain in bone density was observed. In the mice treated with anti-mRANKL Ab intracortical lytic lesions disappeared for the progressive increase of the bone density. The FD-like tissue in untreated (**b** and **c**) and ZA-treated (**e** and **f**) mice consisted of bone trabeculae (*bt*) separated by a cellular fibrous tissue (*ft*). Compared to the untreated mice, the FD-like tissue in the tail vertebrae of mice treated with ZA showed a greater number of osteoclasts, some of which were apoptotic (arrowheads in **f**) or of very large size (“giant osteoclasts”, arrows in **f**). The amount of FD-like tissue was markedly reduced in the tail vertebrae of mice treated with anti-mRANKL Ab (**h** and **i**) in which the marrow cavity was partially obliterated by the newly formed bone and osteoclasts were extremely rare. The inserts in **c**, **f** and **i** show the TRAP positivity (red staining) of the osteoclasts. Panels in **b**, **c**, **e**, **f**, **h** and **i**: H&E. Bars: 250 μm in **b**, **e** and **h**; 100 μm in **c**, **f** and **i**; 40 μm in the inserts

In untreated mice (Fig. 1a) and in mice treated with ZA (Fig. 1d), the radiographic phenotype rapidly progressed with enlargement of cortical lytic areas and appearance of new lytic foci and bone deformities. Lytic lesions appeared also in vertebrae that were not affected at the beginning of the experiments. However, in some vertebrae of ZA-treated mice, a subtle gain in bone density and more clearly differentiated lytic/sclerotic (lucent/opaque) regions were observed along time. In contrast, in mice receiving the anti-mRANKL Ab (Fig. 1g), cortical lytic areas in the tail vertebrae were almost no longer detectable at the radiographic analysis performed at T21. At this time, the radiodensity of the tail vertebrae was increased compared to the pretreatment stage and further increased during the following three weeks. As previously described [17], in the anti-mRANKL Ab group, the final radiographic aspect was variable and strictly related to the initial burden of the disease in each individual vertebra, since the lesions that were more severe in the pretreatment stage showed the greater increase in the radiopacity at the time of sacrifice.

### Histology, TRAP Histochemistry and Histomorphometry

**Fig. 2:**
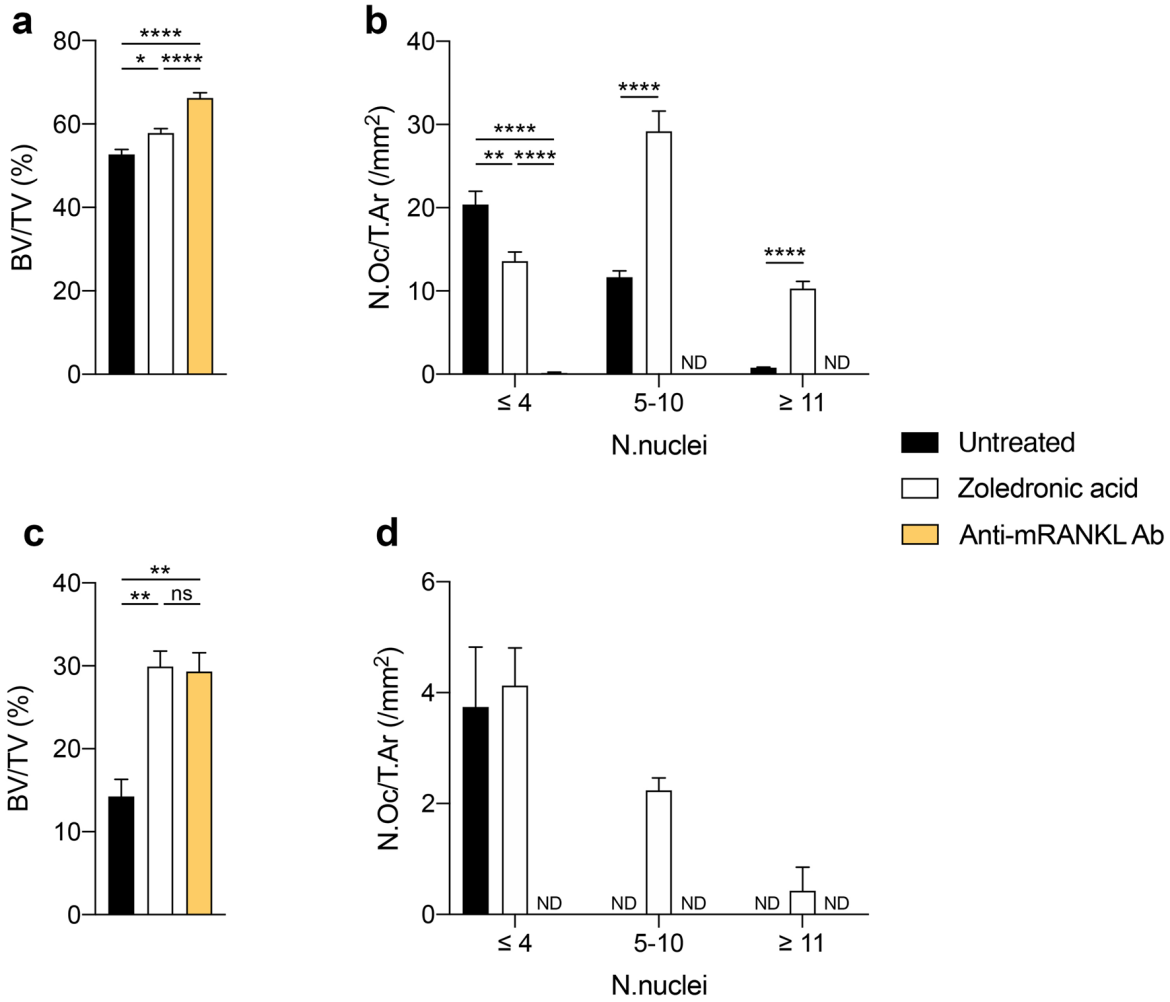
Histomorphometry performed on FD-affected tail vertebrae (**a**, **b**) and unaffected tibiae (**c**, **d**) from untreated mice and mice treated with either ZA or anti-mRANKL Ab. In the FD-affected vertebrae, the BV/TV in ZA-treated mice was significantly higher compared to that of untreated mice but did not reach the values detected in anti-mRANKL Ab-treated mice (**a**). The number of osteoclasts with 5–10 nuclei and with at least 11 nuclei was significantly increased by the ZA treatment, whereas no osteoclasts were detected after treatment with the anti-mRANKL Ab (**b**). In the unaffected tibiae, a comparable increase in BV/TV was observed in ZA and anti-mRANKL Ab-treated mice compared to untreated animals (**c**). Osteoclasts with 5–10 nuclei and > 11 nuclei were observed only in ZA-treated mice (**d**). *p < 0.05; **p < 0.01; ****p < 0.0001; ND: not detected

**Fig. 3:**
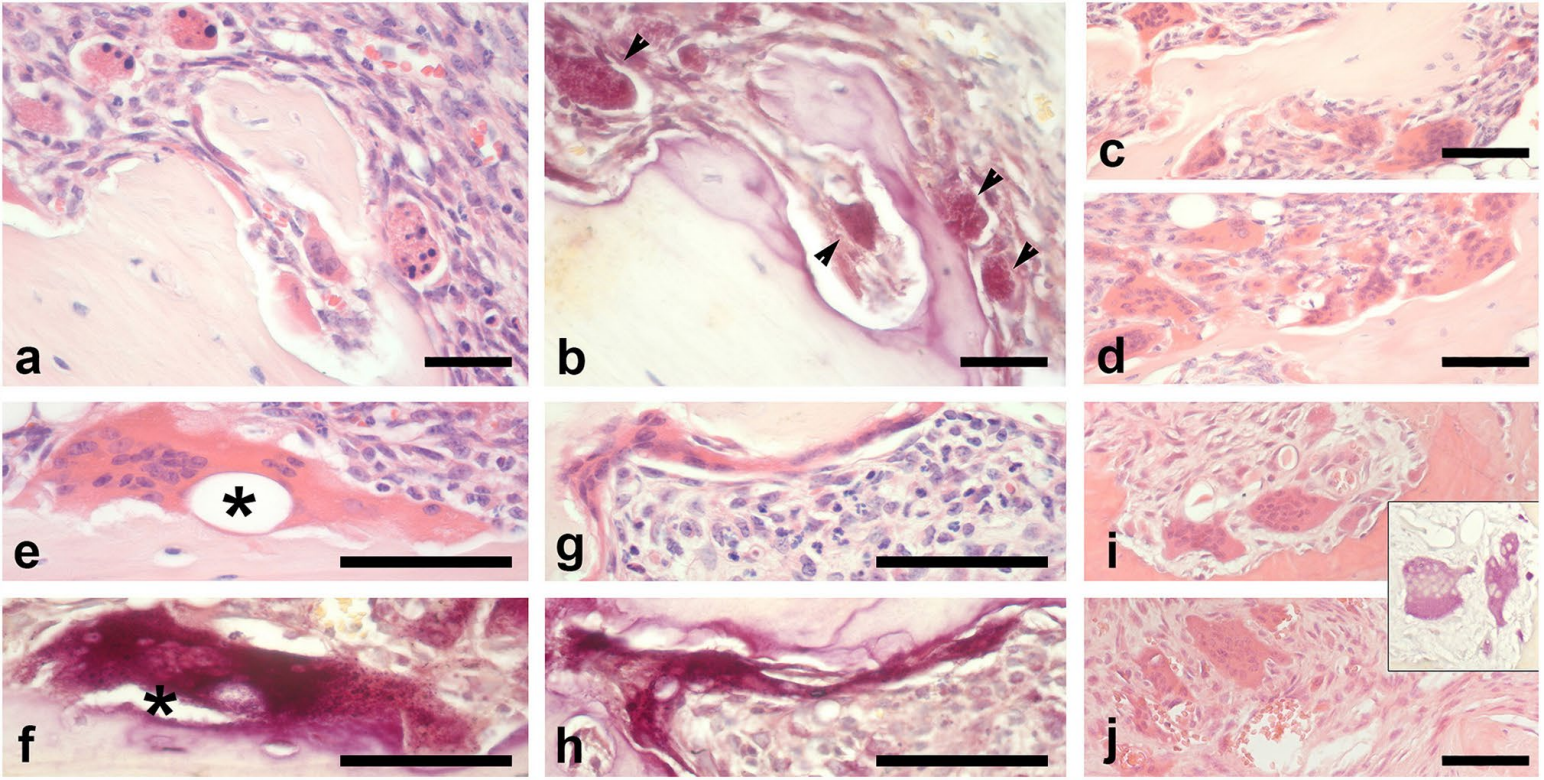
Representative histological images of apoptotic and “giant osteoclasts” in mice treated with ZA are shown in panels (**a**–**h**). The panels **a** and **b** illustrate apoptotic osteoclasts as viewed on serial sections stained with H&E (**a**) and TRAP (**b**). They are easily recognizable for the pyknotic appearance and fragmentation of the nuclei (**a**) and are TRAP-positive (**b**, arrowheads). In panels **c** and **d**, two different low power magnification fields show “giant osteoclasts”. The panels **e**–**h** illustrate two “giant osteoclasts” as viewed on serial sections stained with H&E (**e** and **g**) and for TRAP (**f** and **h**). The “giant osteoclast” illustrated in **e** and **f** shows a very large vacuole (asterisk), while that in **g** and **h** a very aberrant shape, likely reflecting the ZA-related cytoskeletal abnormalities. The panels **i** and **j** illustrate the “giant osteoclasts” in the FD-patient treated with pamidronate [17]. The insert between **i** and **j** illustrates their TRAP staining. Panels in **a**, **c**–**e**, **g**, **i** and **j**: H&E. Bars: 80 μm. The original magnification for panel **i** and for the insert between **i** and **j** is the same of panels **a**–**d** and **j**

As expected based on the radiographic analysis, the histological features of untreated mice (Fig. 1b, c) and ZA-treated mice (Fig. 1e, f) largely overlapped. Both groups showed FD-like areas consisting of fibro-osseous tissue including a variable amount of abnormal bone trabeculae and a large number of TRAP-positive osteoclasts. However, differences in the amount of bone tissue and in osteoclasts were observed. Indeed, in ZA-treated mice, BV/TV was significantly increased (*p* < 0.05, Fig. 2a) compared to untreated mice, confirming the radiographic observation. In the same group, osteoclasts were significantly increased (N.Oc/T.Ar/mm^2^, ZA treated vs untreated, mean ± SEM: 52.89 ± 4.33 vs 32.90 ± 2.53, *p* < 0.0001), frequently apoptotic and “giant”. Based on morphological criteria, apoptotic osteoclasts (Fig. 3a, b) were identified only in the FD lesions of ZA-treated mice (5.62 ± 1.50/mm^2^). The “giant osteoclasts” (Fig. 3c–h) were recognized for the very large size and for the hyper-nucleation (even more than 40) with nuclei variably dispersed throughout the cytoplasm rather than polarized away from the bone surface [3, 4]. Quantitative analysis revealed a number of osteoclasts with 5–10 nuclei and with a number of nuclei ≥ 11 significantly increased compared to untreated mice (Fig. 2b). In addition, they were virtually always devoid of a recognizable lysosome-rich micro-multi-vacuolar zone, occasionally presented a large intra-cytoplasmic vacuole and were very frequently detached from the bone surfaces. As expected from our previous study [17] and consistent with the results of the radiographic analysis, histology of the tail vertebrae of mice treated with anti-mRANKL Ab (Fig. 1h, i) revealed the deposition of new bone within the affected vertebrae, the amount of which varied according to the radiographic phenotype at the beginning of treatment and was significantly higher compared to ZA-treated mice (Fig. 2a).

At FD-unaffected proximal tibia, a significant increase in BV/TV was observed in ZA- and anti-mRANKL Ab-treated mice compared to untreated animals (*p* < 0.01, Fig. 2c) in the absence of a significant difference between the two groups. Osteoclasts with at least 5 nuclei were observed only in the tibiae of the ZA-treated group (Fig. 2d).

### Human FD

As in mice treated with ZA, the human samples revealed the presence of a large amount of intra-lesional TRAP-positive “giant osteoclasts” (Fig. 3i, j).

## Discussion

This study demonstrates that ZA, in agreement with previous reports on the use of N-BPs in FD patients [9, 13, 18] did not affect the pathology and progression of FD lesions in a mouse model of the human disease, in spite of the high dose that was administrated. In contrast, the anti-mRANKL Ab reproduced the beneficial effects previously observed [17] even with only one injection every three weeks.

Histologically, ZA treatment was associated with an increased number of osteoclasts, with osteoclast apoptosis and with the appearance of “giant osteoclasts”.

The lack of significant effect of BPs on FD tissue in both humans [9] and mice (this study) might result, at least in part, from the mineralization deficit of the FD bone, a well-recognized feature of the disease in humans and also in our transgenic mouse model [6, 16–18]. It is well established that BPs exert their function following binding and incorporation into the mineralized bone matrix [2]. Hence, it can be assumed that the amount of BPs incorporated in the osteomalacic bone matrix of FD and, consequently, their availability in the FD microenvironment are locally insufficient to interfere significantly with the resorption activity of intra-lesional osteoclasts. This might reasonably explain why, particularly in pediatric patients, treatment with BPs rarely, if ever, has a beneficial effect on the radiographic appearance and growth of well-established FD lesions and why it is not efficacious in preventing bone fractures.

Nevertheless, the amount of N-BPs that is incorporated in the FD bone and released in the local microenvironment during osteoclast resorption in mouse and humans is sufficient to induce some osteoclast apoptosis as well as the appearance of numerous “giant osteoclasts”. The formation of “giant osteoclasts” upon N-BPs administration is known to occur in different bone diseases [3, 4]. Thus, the phenomenon is not related to the clinical condition for which N-BPs are administrated but rather to their specific mechanism of action [3]. N-BPs inhibit bone resorption by interfering with farnesyl pyrophosphate synthase in the mevalonate pathway, which results in the lack of prenylation of small guanosine triphosphate binding proteins whose appropriate activity is required for function and survival of osteoclasts [2]. As a consequence, the osteoclastic ruffled border does not form, the cytoskeleton disrupts, and cells lose their orientation, detach from the bone surfaces and, eventually, die. Normally osteoclast apoptosis is induced by the high extracellular concentration of calcium released from the bone matrix during resorption [3]. By interfering with bone resorption, N-BPs effectively diminish the calcium release thus prolonging osteoclast lifespan and allowing time for fusion of osteoclasts with additional mononuclear progenitors and formation of giant polykaryons [3]. In FD, as suggested by our results, the formation of “giant osteoclasts” as well as the delay of osteoclast death, might be further favored by the histopathology of the disease since the extracellular concentration of calcium in FD under treatment with N-BPs is likely lower as compared to what expected in conditions in which bone is properly mineralized.

This study also confirmed that inhibition of RANKL, and therefore of osteoclast formation, in EF1α-Gsα^R201C^ mice causes the deposition of mineralized bone matrix at the expenses of the fibro-dysplastic tissue within the FD-like lesions, as we recently reported [17]. This effect was not reproduced in the same transgenic mouse model following treatment with ZA, neither it was ever observed in the FD tissue from patients under treatment with N-BPs. Since the main difference between ZA and anti-mRANKL Ab treatments is the persistence of osteoclasts, it can be hypothesized that in FD osteoclasts influence the differentiation and function of osteogenic cells irrespective of their resorptive activity. The “giant osteoclasts” might give a substantial contribution to this effect. Although their biological properties remain to be addressed, as normal osteoclasts, the large polykaryons contain TRAP and likely the same molecular and vesicular apparatus involved in the physiological or pathological cross-talk between bone resorbing and bone forming cells [21, 22]. Thus, if on the one side the formation of “giant osteoclasts” in FD may represent a histological hallmark of treatment with N-BPs, as in other bone diseases, on the other side it may contribute to explain the lack of a substantial effect of these drugs on the tissue pathology and, more in general, on the natural history of the disease.

Finally, while clearly demonstrating the different consequences of N-BPs and anti-mRANKL Ab on the histology of FD, our current and previous works [17] also suggest a potential usefulness for their association. Based on the deposition of highly mineralized bone matrix during RANKL inhibition, it can be reasonably hypothesized that if administered along with, or immediately after, the anti-mRANKL Ab, N-BPs would effectively bind to the newly formed bone and make it resistant to resorption at the end of the treatment. This approach would also prevent the changes in the serum level of calcium, phosphorus and bone turn-over markers that are well documented in FD patients [5] and are reproduced in our FD mouse model [17] when RANKL inhibition is withdrawn. Studies on the safety and efficacy of this combined therapy are currently in progress in our laboratory.
